# Enzymatic Hydrolysis of Pulse Proteins as a Tool to Improve Techno-Functional Properties

**DOI:** 10.3390/foods11091307

**Published:** 2022-04-29

**Authors:** Martin Vogelsang-O’Dwyer, Aylin W. Sahin, Elke K. Arendt, Emanuele Zannini

**Affiliations:** 1 School of Food and Nutritional Sciences, University College Cork, T12 YN60 Cork, Ireland; m.vogelsangodwyer@umail.ucc.ie (M.V.-O.); aylin.sahin@ucc.ie (A.W.S.); e.zannini@ucc.ie (E.Z.); 2 APC Microbiome Ireland, University College Cork, T12 YT20 Cork, Ireland

**Keywords:** pulse proteins, enzymatic hydrolysis, hydrolysate, protease, functional properties, plant protein

## Abstract

Pulse proteins are being increasingly investigated as nutritious and functional ingredients which could provide alternatives to animal proteins; however, pulse protein ingredients do not always meet the functionality requirements necessary for various applications. Consequently, enzymatic hydrolysis can be employed as a means of improving functional properties such as solubility, emulsifying, foaming, and gelling properties. This review aims to examine the current literature regarding modification of these properties with enzymatic hydrolysis. The effects of enzymatic hydrolysis on the functionality of pulse proteins generally varies considerably based on the enzyme, substrate, processing steps such as heat treatment, degree of hydrolysis, and pH. Differences in protease specificity as well as protein structure allow for a wide variety of peptide mixtures to be generated, with varying hydrophobic and electrostatic properties. Typically, the most significant improvements are seen when the original protein ingredient has poor initial functionality. Solubility is usually improved in the mildly acidic range, which may also correspond with improved foaming and emulsifying properties. More work should be carried out on the potential of enzymatic hydrolysis to modify gelation properties of pulse proteins, as the literature is currently lacking. Overall, careful selection of proteases and control of hydrolysis will be necessary to maximize the potential of enzymatic hydrolysis as a tool to improve pulse protein functionality and broaden the range of potential applications.

## 1. Introduction

There is currently a need to accelerate the development and utilisation of plant-based protein sources, with the end goal of providing alternatives to traditional animal-derived foods. Growing global population and protein demand, awareness of the negative environmental consequences of animal-based food production, as well as ethical and health concerns, are contributing to the increasing interest in the development of plant-based foods, and it has become clear that a dietary transition away from animal protein is needed for sustainability and food security [1,2]. It is now recognised that growing protein-rich plant crops for animal feed is in many cases less efficient and sustainable than direct consumption of plant proteins by humans [3], which incentivises further development and exploitation of plant protein sources, such as pulses. Pulses are leguminous seeds including various peas, beans, chickpeas, lentils, and lupins, generally considered separately from oilseed legumes such as soybeans and peanuts [4,5]. They are typically starch-rich crops with a relatively high protein content, although they are usually lower in protein than soybeans. The dominant protein fractions in pulses are globulins (salt soluble proteins) and albumins (water soluble proteins). Typically, globulins are present in higher amounts than albumins; however, the relative amounts can vary considerably between different pulses, and also due to variety and cultivation conditions, and the albumin/globulin ratio has been reported as high as ~0.5 [6,7,8,9]. The albumins are mainly composed of metabolic proteins and enzymes, and pea albumins include PA-2, PA-1, lipoxygenase, protease inhibitors, and lectins [7,8]. Globulins, on the other hand, are comprised of storage proteins. The two main globulin fractions in pea and other pulse proteins are referred to as legumin and vicilin, and a third fraction, convicilin, may also be present. In general, proteins from different pulses show structural similarities. Legumin is a hexamer with a molecular weight of ~340–360 kDa, whereas vicilin is a trimer with a molecular weight of ~175–180 kDa [10]. Different structural and surface properties of legumin and vicilin can correspond to differences in functionality (e.g., solubility and emulsifying properties); therefore, the legumin:vicilin ratio, which can vary considerably between different pulses and varieties, is an important consideration [11,12]. In addition, the protein composition can be altered with processing, for example, some removal of albumins is likely during isoelectric precipitation [4].

Pulses are increasingly being explored as a nutritious and sustainable source of plant protein. The protein content for most pulses is in the range of ~15–30% of dry matter [13]. This could be considered relatively high (e.g., compared to cereals); however, concentration/isolation steps are required to produce high protein ingredients [14]. Dry processing by milling and air classifying can be used to produce protein concentrates with protein content up to ~70%, depending on the pulse used [15]. Protein isolates with higher protein content (often > 80%) can be produced using aqueous extraction followed by techniques such as isoelectric precipitation or ultrafiltration [16,17]. Pulse protein isolates and concentrates have generated much interest due to their good techno-functional properties. Pea protein ingredients are important in the food industry and are used in a variety of plant-based products, whereas other pulse protein sources are receiving increasing attention for their potential (e.g., faba bean and lentil) [18,19,20,21]. Pulse proteins have shown good promise in plant-based alternatives, and could potentially prove to be useful alternatives for milk, egg, and meat protein, as well as soy protein. Examples include milk alternatives produced with pea, lupin, or lentil protein, as well as meat alternatives produced with pea or faba bean protein concentrate [17,22,23,24].

Depending on the application, certain functional properties may be required, such as solubility, emulsifying, foaming, and gelling ability, or a combination of these. Wide variability in these properties has been observed depending on various factors such as protein source, processing, and environmental conditions [10,16]. In addition, due to differences in structure, it remains challenging to replicate the functionality of animal proteins with plant-derived proteins. For example, the fibrous structure of muscle tissue cannot be easily mimicked using globular plant proteins, and also the unique ‘random coil’ structure of caseins and the casein micelle structure are important for the textural properties of dairy products such as cheese and yoghurt [25]. When formulating plant-based products there is often a gap between the required functionality versus the functionality provided by protein ingredients. Furthermore, the solubility of pulse proteins is particularly poor under mildly acidic conditions, in the pH region near their isoelectric point, as their solubility is typically influenced to a large degree by electrostatic repulsion [10]. Partial enzymatic hydrolysis is a method which has in many cases been shown to improve solubility and other techno-functional properties of proteins, especially in cases where the proteins showed poor functionality to begin with [26,27].

In addition, hydrolysis can also potentially provide the benefit of improved digestibility, for example, enzymatic hydrolysis of lentil protein was found to increase in vitro protein digestibility [28]. Enzymatic hydrolysis is often preferred to chemical hydrolysis as it does not require harsh conditions, is easier to control, and retains the nutritional quality of the protein [29]. As pulse protein ingredients become more widely available and utilised in the food industry, knowledge of the tools and strategies to improve their functionality will be essential in order to broaden the range of applications; therefore, this review aims to focus on current knowledge of the effects of enzymatic hydrolysis on important techno-functional properties of high protein ingredients from pulses, and its potential for improving these properties. Currently, the literature regarding the influence of enzymatic hydrolysis on pulse protein techno-functional properties has not been reviewed. As high-protein pulse ingredients are growing in interest, importance, and variety, it will be important to assess and improve our understanding of techniques such as enzymatic modification.

## 2. Proteases

Proteases (peptidases) are enzymes that cleave peptides and proteins in the presence of water by hydrolysis. Proteases may be classified in various ways. Based on positional specificity, they are divided into two main groups, endo- and exopeptidases. Endopeptidases act on internal bonds of polypeptides, whereas exopeptidases cleave near the ends at the C- or N-terminus; thus, endopeptidases cleave proteins to peptides of various sizes, whereas exopeptidases liberate either a single amino acid residue, a dipeptide or a tripeptide, depending on the type [30,31]. In addition, proteases are classified according to the main chemical group responsible for catalysis at the catalytic site. They include serine proteases, cysteine proteases, threonine proteases, aspartic proteases, glutamic proteases and metalloproteases [30,31]. Furthermore, proteases may be classified according to their origin (i.e., microbial, plant, or animal derived). The majority of industrially used enzymes are of microbial origin [30], and microbially derived alternatives are now available for some traditionally animal-derived proteases [32].

**Figure 1:**
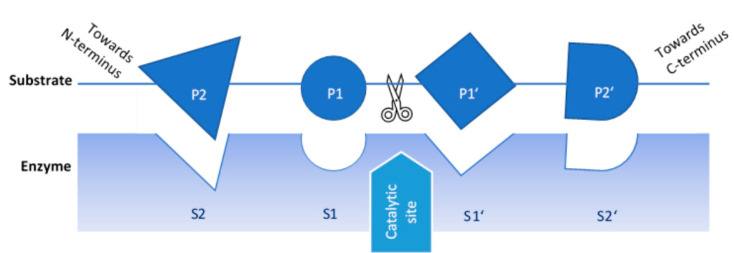
Schematic representation of protease sequence specificity. Adapted from Rawlings and Barrett [31], permission obtained.

Importantly, proteases exhibit sequence specificity, exhibiting a preference for specific amino acids next to the peptide bond to be hydrolysed, corresponding to the amino acid sequence near the enzyme’s catalytic site [31]. This is shown schematically in Figure 1. Amino acid residues at the catalytic site of the protease correspond to specific amino acids in the protein substrate, in each case labelled according to their proximity to the peptide bond to be hydrolysed, and directionally towards the C- or N-terminus; therefore, wide variability in peptides generated can be expected with different enzyme and substrate combinations. In addition, some proteases exhibit broad specificity, whereas others show narrower specificity [28]. Although a protease may be able to hydrolyse multiple peptide bonds, the rate of cleavage may be very different depending on the specific bond [33]. Various food-grade proteases have been utilised to produce hydrolysates of pulse proteins, examples of which are shown in Table 1. Endoproteases are typically used to produce protein hydrolysates, sometimes in combination with exoproteases. Commercial enzyme preparations may contain mainly one protease, or a mixture of proteases. Alcalase is an example of a commonly used serine endoprotease, with broad specificity. It has been well studied and is mainly composed of Subtilisin A (Subtilisin Carlsberg), originating from *Bacillus lichenformis* [34]. Other serine endoproteases include Savinase, trypsin, and chymotrypsin. Trypsin shows narrowly defined specificity and cleaves next to lysine and arginine, whereas chymotrypsin is non-specific, although it preferentially hydrolyses next to certain amino acids, including tryptophan, tyrosine, phenylalanine, and leucine. Neutrase is an example of a zinc metalloprotease, derived from *Bacillus amyloliquefaciens* [35]. Papain and bromelain are cysteine endoproteases derived from papaya latex and pineapple stem, respectively [35]. Flavourzyme is a widely used exoprotease preparation, an enzyme mixture originating from *Aspergillus oryzae*. It contains various exopeptidases and endopeptidases [29]; however, the key enzyme activity according to the manufacturer is that of aminopeptidase, liberating amino acids from the N-terminal. As the name suggests, a major function of Flavourzyme is to improve sensory characteristics, although it has also been shown to modify techno-functional properties [35,36].

Certain environmental conditions are required for effective hydrolysis depending on the protease. In particular, each protease demonstrates temperature and pH optima, as well as a range for each in which the protease is active [26]. Above a certain temperature, denaturation will occur, deactivating the enzyme. Protease activity is sensitive to pH, due to the functional groups involved in the hydrolysis reaction. Generally, serine proteases show highest activity at alkaline pH, cysteine proteases around neutral pH, and aspartic proteases acidic pH [33]. Additionally, protease selectivity should be considered. The rate of hydrolysis of a specific cleavage site can be influenced by various factors, including other amino acids near the cleavage site, pH, temperature, and accessibility of the cleavage site [37,38]; therefore, hydrolysis conditions (e.g., pH, can influence the hydrolysate properties in addition to the rate of hydrolysis).

## 3. Production of Protein Hydrolysates and Assessment of the Extent of Hydrolysis

There are various ways in which enzymatic hydrolysis can be applied to pulse proteins to improve functionality. Typically, a dispersion of protein isolate or concentrate is prepared, incubated under specific conditions with protease(s), and then dried to produce a protein hydrolysate powder [39,40]. Other approaches are also possible, such as incorporating an enzymatic hydrolysis step during food product production or during protein extraction from seed material. Due to the high cost of enzymes, immobilisation methods for enzymes have also been developed, which allows them to be recovered after hydrolysis [41,42]. In addition to batch processes, continuous methods have been developed which allow for lower costs and decreased product variability [43]. Generally, in laboratory-scale studies, a protease is added to a protein dispersion at a specific dosage, and hydrolysis is carried out with controlled temperature and pH, until a specific time or degree of hydrolysis has been reached [44,45]. If pH is not controlled, changes in pH may occur during hydrolysis, depending on the initial pH environment. If pH is above the pKa of the amino groups, newly released carboxyl groups and amino groups will both be deprotonated, with the net effect of releasing protons and lowering pH, whereas if the pH is below the pKa of the carboxyl groups, both the amino and carboxyl groups will be protonated; therefore consuming protons, with the effect of raising pH [27]. After the required hydrolysis duration, the enzyme is usually deactivated by denaturation with a heat treatment step. At laboratory scale, the hydrolysate is typically freeze dried prior to analysis, although this is not always the case. Other steps can include centrifugation (e.g., in some cases, the hydrolysate is centrifuged and only the soluble fraction is recovered) [46]. Such differences in processes should be taken into account as they may have a significant influence on the structural and functional properties of the hydrolysates; with centrifugation, a certain fraction of the proteins/peptides would be excluded, and functionality may also be affected by the drying method [47]. The requirement for enzyme deactivation (typically by heat treatment) is an important disadvantage of enzymatic hydrolysis, due to the harsh conditions and extra energy input required. It is important to consider the effects of the heat-treatment step on protein properties, as structural changes such as unfolding and protein–protein aggregation may influence functionality [48,49]. Many studies make comparisons between hydrolysates and an untreated protein ingredient; however, this does not account for the enzyme deactivation heating step, and significant functional differences have been found between untreated protein isolates/concentrates, and those which have been subjected to the same conditions as the hydrolysates but without the addition of enzymes [45,50]. Additionally, pre-treatments can be applied, which can influence proteolysis, and potentially modify the functionality of hydrolysates. One potential method is initial heat treatment before hydrolysis to induce unfolding of proteins and expose previously buried peptide bonds [26,33]. High-pressure processing has also been explored as a pre-treatment. Al-Ruwaih et al. [51] and Ahmed et al. [52] used this method before the hydrolysis of kidney bean and lentil protein hydrolysates, respectively, resulting in significant differences in functional properties of the hydrolysates.

The degree of hydrolysis, defined as the percentage of peptide bonds hydrolysed relative to the untreated protein substrate, is commonly used to measure the extent of enzymatic hydrolysis; however, there is no standard method for degree of hydrolysis, and the different techniques that are commonly used can yield varying results; therefore, a direct comparison between studies is usually not possible. In addition, some methods may be more suitable for particular substrates or protease types [53]. The various methods and the principles behind them have been reviewed by Rutherfurd [54]. The methods that are mainly used are based on various principles, including base consumption needed to maintain pH (pH-stat method), changes in osmolality (osmometric method), determination of free amino groups (o-phthalaldehyde (OPA) method, trinitrobenzenesulfonic acid (TNBS) method, and formol titration method), and solubility of amino acids and small peptides in trichloroacetic acid (soluble nitrogen-TCA method). In general, measuring the degree of hydrolysis is helpful, as differences in functionality are often found depending on degree of hydrolysis (e.g., a particular functionality might be increased up to a certain degree of hydrolysis, but then decrease on further hydrolysis); however, the degree of hydrolysis alone does not provide specific information on structural changes [27]; therefore electrophoresis, most often in the form of SDS-PAGE, is usually used to gain more specific information on the degradation of proteins during hydrolysis. This allows the approximate molecular weight distribution to be visualised, showing the extent of degradation for different protein fractions along with the appearance of smaller peptides within a certain range. Electrophoresis is particularly useful not just for showing the overall extent of degradation, but also differences in molecular weight distribution, which can provide key information regarding the specificity of the proteases in relation to different protein fractions [35,50]. In addition, size exclusion chromatography can be used to assess peptide size distribution, and is capable of detecting smaller peptides which fall below the sizing range of electrophoresis. Furthermore, liquid chromatography followed by mass spectrometry can be used to separate and identify peptide fractions.

## 4. Solubility

Solubility is usually considered to be a critical functional property of protein ingredients. Many food applications require high solubility, and the ability of proteins to contribute other functionalities such as foaming, emulsifying, and gelling is typically dependent on their initial solubilisation [42,55,56]. Solubility is also important for high protein beverages such as milk alternatives, where sedimentation of insoluble protein particles may be undesirable [24]. One of the disadvantages of plant proteins in general is poor solubility, especially compared with animal proteins such as whey or egg proteins. This can limit the ability of the proteins to act as functional ingredients. Pulse proteins often exhibit better solubility around neutral pH compared to other plant proteins, such as cereal proteins [56]; however, they are generally poorly soluble in the mildly acidic range, near the isoelectric points of the main protein fractions [14,45]. Above the isoelectric point, proteins carry a net negative charge, while they carry a net positive charge below their isoelectric point. The repulsive forces between similarly charged proteins is an important factor for protein solubilisation. Near the isoelectric point, the net charge is negligible and the proteins are prone to precipitation. This generally narrows the range of suitable applications, and even near neutral pH (away from the isoelectric point), pulse proteins may be inadequately soluble in some cases. It has been suggested that commercial protein isolates often demonstrate relatively poor solubility compared with those produced at laboratory scale, attributable to denaturation during processing [57,58].

The solubility of proteins depends on the balance of protein–protein and protein–water interactions, including repulsive and attractive forces. Native globular proteins are typically folded in a conformation where more hydrophobic regions are buried at the centre, whereas more hydrophilic regions are exposed at the surface. Protein structure, and the proportion of polar and non-polar groups exposed to the surface, governs solubility in a given environment [25,59]. Repulsion due to similarly charged proteins promotes solubility, whereas hydrophobic interactions between proteins promotes aggregation and lower solubility [6,27,55]. Both intrinsic and extrinsic environmental factors influence solubility [60]. Protein solubility is usually assessed by centrifuging a protein dispersion, measuring the protein concentration of the supernatant, and expressing it as a percentage of the initial dispersion concentration. It can be difficult to compare directly between studies due to differences in methods, including centrifugation conditions [61]. Aside from the protein’s intrinsic structural properties, the dispersion preparation method/conditions (e.g., homogenisation vs stirring) can have a major impact on solubility values that should not be overlooked [24,62]. Enzymatic hydrolysis generates a variety of smaller peptides, decreasing molecular weight, and at the same increasing the exposure of both hydrophobic regions and ionisable groups. These structural changes often lead to differences in solubility upon hydrolysis [26,27].

**Figure 2:**
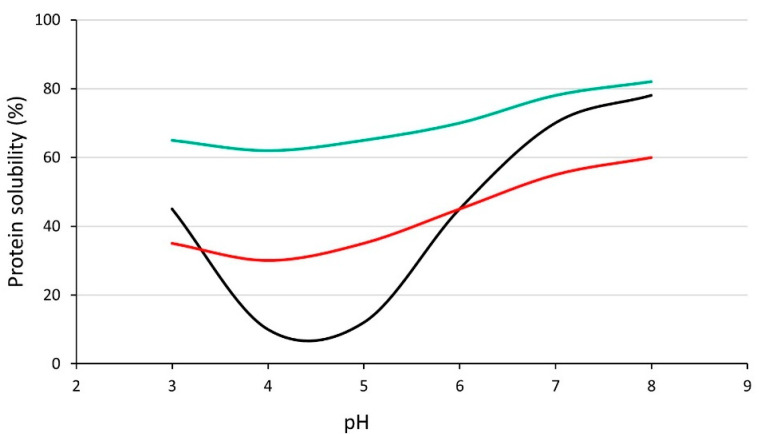
Generalised pH dependent solubility curves showing a typical solubility profile for non-hydrolysed pulse protein and two potential profiles for solubility after hydrolysis. Black: typical pattern for non-hydrolysed pulse proteins; red: hydrolysate with improved solubility near isoelectric point but otherwise reduced solubility; green: hydrolysate with improved solubility across the pH range.

Table 2 shows an overview of the effects of enzymatic hydrolysis on the solubility of pulse protein isolates and concentrates at various pH values. Although the results vary considerably, in most cases, increased solubility is seen in the mildly acidic range near the isoelectric point, whereas outside this range, solubility may increase, but a decrease is also often observed. A typical ‘u-shaped’ pH-dependent solubility curve for pulse proteins is shown in Figure 2, along with two different solubility profiles, which might be expected for hydrolysates. The effect of enzymatic hydrolysis on solubility on a given protein ingredient may vary depending on different factors, including the protease, time/degree of hydrolysis, and environmental conditions. In addition, for the same protease, differences can be seen between substrates (e.g., different pulse types or different varieties); thus, a wide variety of outcomes may be expected with different enzyme and substrate combinations, as well as other factors, such as hydrolysis time and environmental conditions. Differences are often observed based on degree of hydrolysis. Mokni Ghribi et al. [46] found that solubility of chickpea protein treated with Alcalase increased with an increasing degree of hydrolysis across a broad pH range. Betancur-Ancona et al. [63] observed a similar trend with *Phaseolus lunatus* hydrolysates produced with Alcalase or Flavourzyme. In contrast, other studies have found more varied effects, with the increasing degree of hydrolysis not necessarily accompanied by an increase in solubility [35,42,45]. For a given protein substrate and conditions, choice of protease is important if maximum solubility is desirable.

As previously mentioned, changes in solubility have been attributed to decreased molecular weight and an increase in both hydrophobic patches and ionisable groups. Due to differences in specificity between proteases, the hydrolysis products for a given substrate can be very different with regard to these properties [59]. García Arteaga et al. [35] compared the impact of hydrolysis with 11 different proteases on the solubility of pea protein isolate and found major differences depending on the protease applied. At pH 4.5 the solubility of the original isolate was very low at 2%. The least effective protease was found to be chymotrypsin, with little or no improvement at 15 or 30 min hydrolysis. The most effective was Esperase after 120 min hydrolysis, increasing solubility to 71%. At neutral pH, solubility decreased from 51% for the untreated isolate to as low as 24% depending on hydrolysis time with Flavourzyme or chymotrypsin, whereas solubility of 78% was reached with 120 min hydrolysis with Esperase. SDS-PAGE revealed some major differences in molecular weight distribution between the hydrolysates of different proteases, illustrating the differences in specificity leading to peptide mixtures with varying solubility. The study of Barac et al. [50] showed considerable variability in solubility of pea protein isolate with different combinations of pea variety, protease, hydrolysis time, and pH. With papain treatment in particular, major differences in solubility were found between hydrolysates of the two pea varieties tested (L1 and Maja). The authors attributed this to differences in legumin and vicilin content between the varieties, as papain preferentially targeted vicilin and acidic subunits of legumin. The lower solubility of the Maja hydrolysates was attributed to a higher legumin content, and therefore, more hydrophobic peptides and free sulfhydryl groups which promote the formation of insoluble aggregates.

Several studies have assessed changes in surface properties upon hydrolysis of pulse proteins, including surface hydrophobicity, and surface charge (zeta-potential). Surface charge is important as electrostatic repulsion promotes solubility of proteins. At the same time, increased exposure of hydrophobic groups could promote aggregation and reduced solubility. Zhang and Motta [45] found that hydrolysis of the Great Northern bean and navy bean hydrolysates with Alcalase or papain resulted in either increased, unchanged, or decreased surface hydrophobicity at neutral pH; however, the heat-treated control showed higher hydrophobicity compared with the hydrolysates. Interestingly, the solubility of hydrolysates at this pH was not different compared with the untreated protein concentrates. Konieczny et al. [64] hydrolysed pea protein enriched flour with trypsin, Savinase, papain, or pepsin to various degrees of hydrolysis, and found that all hydrolysates had higher surface hydrophobicity and zeta-potential, and lower solubility compared to the untreated ingredient.

It might be expected that hydrolysis should expose previously buried hydrophobic groups, and therefore, higher surface hydrophobicity; however, it can also lead to lower surface hydrophobicity. This has been attributed to aggregation due to hydrophobic interactions, effectively re-burying hydrophobic groups [48]. Hydrolysis has been found to result in more negative surface charge, corresponding to a shift in isoelectric point to slightly lower pH [44,46]; however, compared with intact proteins, the solubility of hydrolysates tends to vary less with changes in pH. Although the impact of enzymatic hydrolysis on pulse protein solubility can vary significantly depending on enzyme and substrate combinations, the greatest increases are usually observed near the isoelectric point. Increased solubility at an acidic pH can be particularly useful for acidic products where high solubility is necessary, for example, faba bean protein hydrolysates have been used to fortify apple juice, in a pH range where the original protein extract was poorly soluble [65].

**Table 2:** Overview of the effects of enzymatic hydrolysis on solubility, with various pulse protein sources and proteases.

Reference	Protein Source	Protease	Effect on Solubility
Barać et al. [66]	Pea protein isolate	Chymosin	Increased at pH 3; increased/no difference at pH 5 depending on HT; decreased at pH 7; increased at pH 8
Barac et al. [50] *	Pea protein isolate (L1)	Papain	Increased at pH 3 and 5; increased/decreased at pH 7 depending on HT; increased at pH 8
		*S. griseus* protease	Increased at pH 3 and 5; decreased at pH 7 and 8
	Pea protein isolate (Maja)	Papain	Increased at pH 3 and 5; increased/decreased at pH 7 depending on HT; decreased at pH 8
		*S. griseus* protease	Increased at pH 3, 5 and 7; increased/decreased at pH 8 depending on HT
Betancur-Ancona et al. [63]	*P. lunatus* protein isolate	Alcalase	Increased at pH 2, 4, 6, 8 and 10
		Flavourzyme	Increased/no difference at pH 2 depending on HT; increased at pH 4 and 6; increased /no difference at pH 8 and 10 depending on HT
Eckert et al. [39]	Faba bean protein isolate	Pepsin	Increased at pH 5 and 7
		Trypsin	Increased at pH 5 and 7
		Flavourzyme	Increased at pH 5 and 7
		Neutrase	Increased at pH 5 and 7
García Arteaga et al. [35]	Pea protein isolate	Alcalase	Increased at pH 4.5; increased/no difference at pH 7 depending on HT
		Papain	Increased at pH 4.5; no difference at pH 7
		Esperase	Increased at pH 4.5 and pH 7
		Bromelain	Increased at pH 4.5; decreased/no difference at pH 7 depending on HT
		Trypsin	Increased at pH 4.5; increased/no difference at pH 7 depending on HT
		Chymotrypsin	Increased/no difference at pH 4.5 depending on HT; decreased at pH 7
Klost and Drusch [44]	Pea protein concentrate	Trypsin	Decreased/no difference at pH 3 depending on DH; increased at pH 4, 5, and 6; decreased at pH 7
Konieczny et al. [64]	Pea protein-enriched flour	Trypsin	Decreased at pH 4, 7 and 10
		Savinase	Decreased at pH 4, 7 and 10
		Papain	Decreased at pH 4, 7 and 10
		Pepsin	Decreased at pH 4, and 7; decreased/no difference at pH 10 depending on DH
Mokni Ghribi et al. [46]	Chickpea protein isolate	Alcalase	Increased at pH 2, 4, 6, 8, 10 and 12
Schlegel et al. [40]	Lupin protein isolate	Alcalase	Increased at pH 4, 5 and 6; no difference at pH 7, 8 and 9
		Papain	Increased at pH 4, 5 and 6; no difference at pH 7, 8 and 9
		Neutrase	Increased at pH 4, 5 and 6; no difference at pH 7, 8 and 9
		Protease N-01	Increased at pH 4 and 5; no difference at pH 6, 7, 8 and 9
		Flavourzyme	Increased at pH 4 and 5; no difference at pH 6; decreased at pH 7, 8 and 9
		Protamex	Increased at pH 4, 5 and 6; no difference at pH 7, 8 and 9
		Corolase 7089	Increased at pH 4, 5 and 6; no difference at pH 7, 8 and 9
		Pepsin	Increased at pH 4, 5 and 6; no difference at pH 7, 8 and 9
		Corolase N	Increased at pH 4, 5 and 6; no difference at pH 7, 8 and 9
Segura-Campos et al. [36]	Cowpea protein concentrate	Alcalase	Decreased at pH 2; increased at pH 4 and 6; decreased at pH 8 and 10
		Flavourzyme	Increased at pH 2, 4, 6, 8 and 10
Xu et al. [67]	Chickpea protein isolate	Alcalase	Increased at pH 2, 4, 7 and 9
		Flavourzyme	Increased at pH 2, 4, 7 and 9
Yust et al. [42]	Chickpea protein isolate	Alcalase	Increased/no difference at pH < 4 depending on DH; increased at pH ~4–6; increased/decreased at pH 7 depending on DH; increased at pH 8, 9 and 10
Zhang and Motta [45] *	Great Northern bean protein concentrate	Alcalase	Decreased/no difference at pH 3 depending on DH; increased at pH 4, 5 and 6; no difference at pH 7
		Papain	Decreased at pH 3; increased at pH 4, 5 and 6; no difference at pH 7
	Navy bean protein concentrate	Alcalase	Increased/no difference at pH 3 depending on DH; decreased/no difference at pH 4 depending on DH; increased at pH 5 and 6; no difference at pH 7
		Papain	Increased at pH 3; no difference at pH 4; increased at pH 5 and 6; no difference at pH 7

This table is intended as an overview only—methodology, data representation, and statistics can vary between studies, making direct comparisons difficult. HT: hydrolysis time; DH: degree of hydrolysis. * Compared with thermally treated control.

## 5. Emulsifying Properties

Many proteins are useful as emulsifiers due to their structure and amphiphilic properties [68]. Various foods consist of oil in water emulsions, such as milk, mayonnaise, and dressings, or water in oil emulsions, such as margarine. Ideally, small emulsifier-coated droplets are dispersed in the continuous phase and should be resistant to aggregation and separation. Proteins stabilise emulsions by reducing the interfacial tension between the two immiscible phases, thus lowering the overall free energy [69]. The balance of hydrophobic and hydrophilic properties of proteins are important in determining their effectiveness as emulsifiers. The protein should possess good solubility in water and be capable of rapid migration to, and adsorption at, the oil–water interface during homogenisation [69]. Once at the interface, globular proteins may structurally rearrange in a conformation where more hydrophilic regions extend to the water phase, whereas more hydrophobic regions extend into the oil phase [68,70]. Emulsion stability depends on protein–protein interaction to form a strong viscoelastic layer at the interface. At the same time, electrostatic repulsion is generally important for prevention of droplet aggregation and phase separation. Overall, as well as solubility, an appropriate balance and distribution of hydrophobic and hydrophilic regions is required [68,69].

Pulse proteins such as lentil, lupin, pea, and chickpea have been shown to be useful emulsifiers for various applications, including milk alternatives and salad dressings [24,71,72]; however, enzymatic hydrolysis could potentially be a useful tool to modify emulsifying properties where improvement is required. By decreasing molecular weight and exposing hydrophobic regions, controlled hydrolysis can potentially deliver an improved ability to form and stabilise emulsions [27]. Emulsifying properties of protein ingredients can be examined using various methods. Emulsifying activity and stability indices are often measured using the turbidimetric method of Pearce and Kinsella [73]. Other approaches include measuring the maximum amount of oil capable of being emulsified with a defined protein dispersion before phase inversion [40]. Emulsion stability can be assessed in terms of separation rate or cream layer height [72,74]. In addition, particle size measurements provide useful information on emulsifying properties [44,45]. Caution should be exercised when comparing studies, as there are often major differences in methods of emulsion preparation and composition, as well as analytical methods.

Table 3 shows the effects of hydrolysis on emulsifying properties of various pulse protein ingredients, using various proteases. Somewhat similarly to solubility, the effects can vary considerably with enzyme, substrate, degree of hydrolysis, and pH. Emulsifying properties often improve near the isoelectric point, along with increased solubility, but this is not always the case. Avramenko et al. [48] found that lentil protein hydrolysates produced with trypsin had lower emulsifying activity and emulsion stability indices than the untreated protein, regardless of degree of hydrolysis. At the same time, the hydrolysates had lower surface hydrophobicity (possibly due to aggregation), greater surface charge and lower interfacial tension. It was suggested that the reduced surface hydrophobicity negatively influenced the emulsifying properties. Barac et al. [50] found that the effect of hydrolysis on the emulsifying activity and emulsion stability indices of pea protein was dependent on the protease, pea variety, pH environment, and hydrolysis time. It was suggested that where reductions in emulsifying properties were observed, the formation of high molecular weight inflexible aggregates could be a key factor. García Arteaga et al. [35] found that hydrolysis either improved or had no significant effect on the emulsifying capacity of pea protein, depending on the enzyme. The highest emulsifying capacity was observed for trypsin and chymotrypsin hydrolysates.

Numerous studies show that emulsifying properties of pulse protein hydrolysates can vary considerably according to the degree of hydrolysis [39,45,46,63], and in many cases, the emulsifying properties seem to be more sensitive than solubility to degree of hydrolysis. The formation of small oil droplets and resistance to flocculation/coalescence is important for avoidance of phase separation in oil in water emulsions. Tamm et al. [7] investigated the impact of a trypsin or Alcalase hydrolysis of pea protein concentrate on emulsion characteristics. They found that the Alcalase hydrolysis had a negative effect, especially at higher degrees of hydrolysis where emulsions separated quickly. In contrast, trypsin hydrolysates generally resulted in improved emulsions with increasing degree of hydrolysis, with smaller droplet sizes, stronger interfacial film formation, and higher zeta potential. Klost and Drusch [44] assessed the droplet size and zeta-potential of emulsions stabilised with pea protein concentrate, either untreated or hydrolysed with trypsin, as a function of pH. Especially with higher degree of hydrolysis, they found that the emulsions were less stable when they were away from the isoelectric point compared to the control. Larger droplets were likely due to flocculation, which are also visible in micrographs. Overall, they hypothesised that for the hydrolysates, hydrophobic interactions were dominant over electrostatic repulsion across the pH range. Zhang and Motta [45] prepared hydrolysates of Great Northern bean or navy bean protein concentrate, using Alcalase or papain. They found that emulsions prepared with hydrolysates generally had smaller droplet size compared with those prepared with the untreated concentrates, and for all samples there was little or no increase in droplet size over an 8 day period. For the Alcalase hydrolysates of both the Great Northern bean and navy bean protein, the smallest droplet size was observed with the highest degree of hydrolysis, whereas for the papain hydrolysates, the smallest droplet size was observed for the low and intermediate degree of hydrolysis. Interestingly, heat-treated controls (i.e., non-hydrolysed samples otherwise subjected to the same conditions as the hydrolysates) formed emulsions with smaller droplet sizes compared with those of the untreated ingredients. This also corresponded with higher surface hydrophobicity and lower surface tension, which underlines the fact that processing steps such as heat treatments can significantly impact protein structure and functionality and should not be overlooked.

It is evident that careful control of hydrolysis is often necessary to achieve improved emulsion stability. In addition, heat stability of emulsions is an important and sometimes overlooked consideration, as many products will require a heat treatment step to ensure microbial stability. In one study, hydrolysis of chickpea protein isolate with Alcalase improved emulsion heat stability only at the lowest degree of hydrolysis tested, and otherwise resulted in a considerably lower stability [42]. In a similar study, hydrolysis of chickpea protein isolate with Flavourzyme resulted in a slightly increased or decreased heat stability of emulsions depending on the degree of hydrolysis [41].

In general, increased hydrophobicity resulting from exposure of hydrophobic groups has been recognised as an important factor in improving the emulsifying properties of pulse proteins [45,48,75]. At the same time, this may lead to aggregation and impaired emulsifying ability [48,50]. It is evident that for a given protein ingredient, careful choice of protease and hydrolysis conditions will be necessary in order to generate peptides with the specific properties favouring formation of stable emulsions (i.e., size, amphiphilic properties, and molecular flexibility). As peptides in a certain size range are required to form a stable viscoelastic film at the oil-water interface, excessive hydrolysis can lead to reduced emulsion stability [22,39,43,53]. Moreover, loss of amphiphilicity could occur; therefore, the high variability found in studies is not surprising, due to the very diverse potential for different peptide mixtures. In particular, differences in protein composition (e.g., between different varieties) can have a major influence and should not be overlooked [50].

Another consideration is the type of emulsion product of interest, as different applications may have very different characteristics, and therefore, different emulsification requirements and challenges. For example, salad dressings may have a low protein/oil ratio, acidic pH, and high viscosity, whereas high-protein milk alternatives would likely have a higher protein/oil ratio, neutral pH, and low viscosity. Many studies use fundamental tests to predict functionality which may not always be relevant for specific applications.

**Table 3:** Overview of the effects of enzymatic hydrolysis on emulsifying properties from various protein sources and proteases.

Reference	Protein Source	Protease	Effect on Emulsifying Properties
Ahmed et al. [52]	Lentil protein isolate	Alcalase	EAI: decreased; ESI: decreased
Al-Ruwaih et al. [51]	Kidney bean protein isolate	Alcalase	EAI: increased (but decreased for high pressure treated sample) ESI: decreased
Avramenko et al. [48]	Lentil protein isolate	Trypsin	EAI: decreased; ESI: decreased
Barać et al. [66]	Pea protein isolate	Chymosin	EAI: increased at pH 3; increased/no difference at pH 5 depending on HT; increased/decreased at pH 7 depending on HT; decreased at pH 8 ESI: decreased at pH 3; increased/decreased at pH 5 depending on HT; increased/no difference at pH 7 and 8 depending on HT
Barac et al. [50]	Pea protein isolate (L1)	Papain	EAI: increased at pH 3, 5, 7, and 8 ESI: increased at pH 3; decreased/no difference at pH 5 depending on HT; increased at pH 7 and 8
		*S. griseus* protease	EAI: increased at pH 3, 5, 7 and 8 ESI: increased at pH 3; decreased at pH 5; increased at pH 7 and 8
	Pea protein isolate (Maja)	Papain	EAI: decreased at pH 3; increased/decreased at pH 5 and 7 depending on HT; increased/no difference at pH 8 depending on HT ESI: increased/decreased at pH 3 and 5 depending on HT; decreased at pH 7 and 8
		*S. griseus* protease	EAI: increased/decreased at pH 3, 5, 7, and 8 depending on HTESI: increased at pH 3 and 5; increased/decreased at pH 7 depending on HT; decreased at pH 8
Betancur-Ancona et al. [63]	*P. lunatus* protein isolate	Alcalase	EC: decreased at pH 2, 4, 6, 8, and 10 ES: decreased at pH 2; increased at pH 4; decreased at pH 6, 8, and 10
		Flavourzyme	EC: increased at pH 2; no difference at pH 4; increased/no difference at pH 6 depending on HT; increased at pH 8 and 10ES: No difference at pH 2; increased at pH 4 and 6; decreased/no difference depending on HT at pH 8 and 10
Eckert et al. [39]	Faba bean protein isolate	Pepsin	Decreased EAI and ESI
		Trypsin	Increased/decreased EAI and ESI depending on HT
		Flavourzyme	Decreased EAI; increased ESI
		Neutrase	No difference in EAI, increased ESI
García Arteaga et al. [35]	Pea protein isolate	Alcalase	EC: no difference
		Papain	EC: no difference
		Esperase	EC: increased/no difference depending on HT
		Bromelain	EC: no difference
		Trypsin	EC: increased
		Chymotrypsin	EC: increased
Konieczny et al. [64]	Pea protein-enriched flour	Trypsin	EAI: increased at pH 4; increased/decreased at pH 7 depending on DH; increased at pH 10 ESI: decreased at pH 4, 7, and 10
		Savinase	EAI: increased/decreased at pH 4 depending on DH; decreased at pH 7; increased at pH 10 ESI: decreased at pH 4, 7, and 10
		Papain	EAI: decreased at pH 4, 7, and 10 ESI: increased at pH 4; decreased at pH 7 and 10
		Pepsin	EAI: decreased at pH 4, 7, and 10 ESI: decreased at pH 4, 7, and 10
Mokni Ghribi et al. [46]	Chickpea protein isolate	Alcalase	EAI: increased/decreased depending on DH ESI: decreased/no difference depending on DH
Schlegel et al. [40]	Lupin protein isolate	Alcalase	EC: decreased
		Papain	EC: decreased
		Neutrase	EC: decreased
		Protease N-01	EC: no difference
		Flavourzyme	EC: decreased
		Protamex	EC: decreased
		Corolase 7089	EC: no difference
		Pepsin	EC: no difference
		Corolase N	EC: no difference
Wani et al. [76]	Kidney bean protein isolate (French Yellow)	Papain	EAI: increased/decreased at pH 3 depending on HT; decreased at pH 5; increased at pH 7 ESI: increased/no difference at pH 3 and 5 depending on HT; no difference at pH 7
	Kidney bean protein isolate (Contender)	Papain	EAI: increased at pH 3, 5 and 7 ESI: decreased/no difference at pH 3 depending on HT; increased/no difference 5 depending on HT; decreased at pH 7
	Kidney bean protein isolate (Master Bean)	Papain	EAI: increased at pH 3, 5 and 7 ESI: increased/no difference at pH 3 depending on HT; decreased at pH 5 and 7
	Kidney bean protein isolate (Local Red)	Papain	EAI: increased at pH 3, 5 and 7 ESI: no difference at pH 3; increased/no difference at pH 5 and 7 depending on HT
Wani et al. [77]	Black gram protein isolate (Mash 1-1)	Papain	EAI: increased at pH 3, 5 and 7 ESI: increased/no difference at pH 3 depending on HT; increased at pH 5; decreased/no difference at pH 7 depending on HT
	Black gram protein isolate (PU-19)	Papain	EAI: increased/decreased at pH 3 and 5 depending on HT; increased/no difference at pH 8 depending on HT ESI: increased/decreased at pH 3 and 5 depending on HT; decreased at pH 8
	Black gram protein isolate (T-9)	Papain	EAI: increased/decreased at pH 3 depending on HT; increased at pH 5; increased/decreased at pH 7 depending on HT ESI: increased at pH 3 and 5; increased/no difference at pH 7 depending on HT
Xu et al. [67]	Chickpea protein isolate	Alcalase	EAI: increased; ESI: increased
		Flavourzyme	EAI: increased; ESI: increased

This table is intended as an overview only—methodology, data representation, and statistics can vary between studies, making direct comparisons difficult. HT: hydrolysis time; DH: degree of hydrolysis; EAI: emulsifying activity index; ESI: emulsion stability index; EC: emulsifying capacity; ES: emulsion stability.

## 6. Foaming Properties

Foams can be described as dispersions of gas bubbles, surrounded by a liquid or solid continuous phase [78]. Foam formation and stability are key properties for many food applications, including meringues, cakes, ice cream, frothed milk beverages, whipped toppings, and mousses [2,78,79], many of which involve proteins as surfactants. Proteins can stabilise foams by reducing interfacial tension, aligning and forming a viscoelastic layer at the air–water interface. The molecular properties of proteins required to produce stable foams are somewhat similar to those required for emulsions (e.g., appropriate amphiphilicity, flexibility, solubility and size); therefore, enzymatic hydrolysis is often a useful tool for the modification of foaming properties.

Foaming properties are typically measured in terms of foaming capacity (the amount of foam produced relative to starting volume), and foam stability (the proportion of foam remaining after a specified time). Other characteristics such as foam density and texture may also be of interest. Some pulse proteins already display high foaming capacity and stability, and in such cases enzymatic treatment may not be useful for enhancing these properties; however, others with poor foaming properties might be significantly improved.

Table 4 shows examples of the effect of enzymatic hydrolysis on foaming properties of various pulse proteins. As with emulsifying properties, there can be considerable variability in the effects of hydrolysis on foaming properties depending on enzyme, substrate, degree of hydrolysis, and pH [50]. Hydrolysis can be useful for improving foaming capacity near the isoelectric point, which can be related to increased solubility. For example, Eckert et al. [39] found that the foaming capacity of faba bean protein increased to varying degrees at pH 5 with pepsin, trypsin, Flavourzyme, or Neutrase, whereas at pH 7, foaming capacity was either increased or unchanged depending on the protease and hydrolysis time. At both pH 5 and 7, pepsin hydrolysis for 15 min resulted in the highest foaming capacity. At the same time, decreased foaming capacity may also correspond to decreased solubility after hydrolysis [64]. Similarly to emulsions, at a higher degree of hydrolysis, decreased foam stability may be observed. Even though the peptides may have good solubility and migrate quickly to the air/water interface, they may be too small to form and maintain a strong interfacial film [80]. This was observed in the studies of Ahmed et al. [52] and Al-Ruwaih et al. [51] where Alcalase hydrolysis led to an increased foaming capacity but decreased foam stability for lentil and kidney bean protein, respectively. Betancur-Ancona et al. [63] found that hydrolysis of *Phaseolus lunatus* protein isolate with Flavourzyme increased foaming capacity and foam stability across a range of pH values. Alcalase hydrolysis, on the other hand, resulted in lower foaming capacity across the pH range, which was attributed to a higher DH compared with Flavourzyme, and also decreased or increased stability depending on hydrolysis time and pH.

Overall, similarly to emulsifying properties, enzymatic hydrolysis has great potential for tailoring the foaming properties of pulse protein ingredients, but at the same time, it may be difficult to predict and requires careful optimisation (i.e., choice of protease and hydrolysis conditions). As with emulsification, foam stabilisation also requires peptides with specific properties. There seems to be wide variation in the foaming properties of pulse protein ingredients, thus many are already effective foaming agents, and in those cases, hydrolysis can reduce foaming capacity, and quite often foam stability; however, in some cases, hydrolysis may be very useful for improving these properties, and may prove useful in providing effective alternatives to animal proteins used for foam formation (e.g., egg proteins).

## 7. Gelation and Rheological Properties

Gelation is important for various foods, including processed meats/meat alternatives, cheese, yogurt, tofu, and desserts. There is now considerable interest in formulating plant-based alternatives to products such as meat and cheese, which require certain textural properties that proteins could potentially contribute to [22,25,81]. Pulse proteins can play a functional role in gelled products; however, it can be difficult to mimic the structural and textural properties of the original products. Gelation of proteins can occur when the proteins unfold, allowing interaction to form a three-dimensional crosslinked network capable of binding water. A critical concentration must be reached before gelation can occur. Proteins in gel structures can be linked by both non-covalent (electrostatic, hydrogen bonds, hydrophobic) and covalent interactions (disulphide bonds) [61,78]. Most often, heat-induced gelation is studied; however, gelation may also be induced or aided by other means including pH changes (usually acid gels), changing ionic strength, high-pressure processing, or enzymatic crosslinking [27,78,82].

Many pulse proteins can form gels; however, they can be relatively weak (e.g., when compared to soy protein gels, attributable at least in part to the lower prevalence of sulfhydryl groups, and consequently, fewer disulphide bonds in the final gels) [83,84]. There is relatively little literature available on the impact of enzymatic hydrolysis on the gelation of pulse proteins; however, for other proteins such as whey and soy, various effects have been observed with enzymatic hydrolysis. Hydrolysis can result in increased gel strength, decreased gel strength, or no gel formation, depending on factors such as protease, degree of hydrolysis, and pH [85,86]. In some cases, limited hydrolysis may lead to improved gelling ability. It is possible that exposure of reactive groups during limited hydrolysis could allow for increased protein–protein interaction during heating, and structural changes could alter the type of network formed. At the same time, above a certain degree of hydrolysis, peptide sizes tend to be too small to form a continuous network, and gelation is impeded [27,59].

Felix et al. [87] examined the impact of hydrolysis with trypsin on the heat gelling properties of pea protein concentrate, at pH 2, 6.5, and 8. The mechanical spectra revealed little impact of hydrolysis on gel strength at low degrees of hydrolysis. At higher degrees of hydrolysis, however, gel strength was reduced at pH 8 compared to pH 6.5. All gels were very weak at pH 2 regardless of treatment. Some differences were apparent between samples regarding the type of gel interactions. Different contributions of ionic bonds, hydrogen bonds, hydrophobic interactions, and disulphide bonds could be seen, depending on hydrolysis time as well as pH. Pea protein gel characteristics have been shown to be highly dependent on pH, and to a lesser extent on ionic strength [88]. Klost et al. [89] prepared fermentation-induced gels from pea protein concentrate, and hydrolysates thereof, to investigate the impact of hydrolysis on the gel rheological properties. The hydrolysates were produced with Protamex, trypsin, or Alcalase. The Alcalase treated sample was unable to form a gel due to the low molecular weight of the peptides. Gels prepared with Protamex or trypsin showed very little difference in rheological properties compared to the unhydrolyzed sample; however, hydrolysis did modify the interaction between protein fractions, with trypsin promoting increased involvement of vicilin in the gel structure. Guldiken et al. [84] compared the heat-gelation properties of faba bean, lentil, and yellow pea protein concentrates, and found that gelation properties were influenced by hydrophobicity and legumin:vicilin ratio; therefore, it may be useful to consider enzyme specificity in relation to these properties.

Due to the relatively small amount of literature available on the effect of hydrolysis on pulse protein gelation, it is difficult to grasp an overall picture of its potential. As previously mentioned, pulse protein gels can be relatively weak, and may be more suitable for softer gelled applications (e.g., yogurts, soft cheese alternatives, or desserts). Hydrolysis could potentially be used to alter gel characteristics to improve texture; however, it is clear that the extent of hydrolysis may need to be very limited to avoid impaired network formation. In addition, it may be expected that for protein ingredients with very poor solubility, in some cases, hydrolysis could improve solubility, and therefore, gelation potential.

As well as gelation, enzymatic hydrolysis could be used to modify the rheological properties of pulse proteins in liquid systems. Enzymatic hydrolysis of protein dispersions can often result in decreased viscosity. Hydrolysis with Alcalase was found to reduce the viscosity of lentil protein [52] and kidney bean protein [51] dispersions. Bajaj et al. [90] examined the effect of hydrolysis with various proteases on pea protein dispersions with high initial viscosity, to reduce viscosity and facilitate microencapsulation of flaxseed oil. They found a considerable reduction in viscosity with most of the treatments. Viscosity reduction with enzymatic hydrolysis could be particularly useful for high-viscosity pulse protein ingredients, for example, in nutritional beverage applications where high protein content but low viscosity is required. At the same time, bitterness could present difficulties for such products.

## 8. Sensory Considerations

Although protein ingredients can provide essential functionality to food products, they may be of limited use if they contribute undesirable sensory attributes. One of the key limitations of protein hydrolysates generally, is the generation of bitter peptides; therefore, reduction or elimination of bitterness in hydrolysates has become an important concern for the food industry [91,92]. Bitter peptides can be generated with hydrolysis of many food proteins; however, some are particularly susceptible (e.g., casein) [93]. Much work has focused on dairy and soy proteins hydrolysates; however, bitterness is an important concern for various hydrolysates from various protein sources including pulses. Depending on the type of food product, some level of bitterness may be desirable or acceptable; however, if the level of bitterness is excessive, sensory quality is reduced [93,94,95]. This may be particularly important for high protein beverage applications [91]. As well as peptides, free amino acids can elicit taste, including bitter, umami, sweet, and sour [95].

It is generally accepted that the perception of bitterness is related to the generation of small peptides with a high proportion of hydrophobic side chains [31,89]. Hydrolysis exposes hydrophobic residues which were previously buried in the intact protein [87]. Although these peptides tend to be more hydrophobic, it is difficult to use this property alone to predict the level of bitterness. As well as the proportion of certain amino acid residues, the amino acid sequence, peptide length, and terminal amino acids, affect bitterness level [95,96]. Several important factors which influence the bitterness of protein hydrolysates should be considered. These include the composition and hydrophobicity of the substrate, the protease(s) used, the degree of hydrolysis, any separation steps such as filtration or centrifugation, and other components which could mask bitterness [33].

In sensory evaluations, a bitter substance is typically used as a reference, such as a caffeine solution or a bitter hydrolysate solution. Cho et al. [91] investigated the relationship between peptide properties and the bitterness of two commercial soy protein hydrolysates. They fractionated the hydrolysates based on molecular weight and found for both hydrolysates that the 5–10 kDa fractions had the highest bitterness, with bitterness decreasing towards relatively higher or lower molecular weight fractions. Interestingly, they did not find a correlation between hydrophobicity of the fractions (based on amino acid composition) and bitterness.

Various studies have shown that bitterness is influenced by the protease used and degree of hydrolysis. Seo et al. [97] used taste dilution analysis (taste threshold concentration) to assess differences in bitterness in soy protein hydrolysates. At a constant degree of hydrolysis, they found the highest bitterness for Alcalase hydrolysate, and the lowest for Flavourzyme hydrolysate. Intermediate values were found for Neutrase, Protamex, papain, and bromelain. This illustrates the importance of protease specificity in relation to the peptide mixture produced and corresponding bitterness, for a given substrate. Humiski and Aluko [92] compared the bitterness of pea protein hydrolysates produced with different proteases. They found the highest bitterness for the hydrolysate produced with Alcalase, followed by Flavourzyme, trypsin, chymotrypsin, and papain hydrolysates. The authors suggested that the higher bitterness of the Alcalase hydrolysate could be related to its broad specificity and preference for cleaving near hydrophobic residues and that the release of free amino acids by Flavourzyme could have contributed to increased bitterness. García Arteaga et al. [35] measured bitterness of pea protein isolate hydrolysed with various protease preparations, with a hydrolysis time of 15 min or 120 min. Significantly higher bitterness was found, compared with the unhydrolysed protein isolate for Alcalase at 15 min hydrolysis, Esperase at 120 min hydrolysis, and Savinase at 15 min and 120 min hydrolysis. This also corresponded with a relatively higher degree of hydrolysis for these proteases, which are known to exhibit broad specificity. No significant differences were found compared with the protein isolate for the other proteases, which included Flavourzyme, Neutrase, Protamex, trypsin, chymotrypsin, papain, bromelain, and Corolase 7089. Schlegel et al. [40] compared sensory properties of lupin protein hydrolysates using nine different protease preparations. The hydrolysate prepared with Alcalase was rated as being extremely bitter, and was the only hydrolysate for which bitterness was significantly higher than the untreated protein isolate. In a similar study, Meinlschmidt et al. [98] assessed the bitterness of soy protein hydrolysates produced with various proteases and different hydrolysis times. The intensity of bitterness varied with the protease as well as hydrolysis time. Alcalase and Corolase 2TS hydrolysates had a significantly higher bitterness compared with the protein isolate at all hydrolysis times, whereas for most of the other proteases, a significantly higher bitterness was only found for certain hydrolysis times. Generally, it was found that the relationship between hydrolysis time and bitterness varied with the protease; for example, with Neutrase, bitterness was only significant after longer periods of hydrolysis, whereas with papain, hydrolysates had a significantly higher bitterness compared with the protein isolate at 10 and 30 min, but not at 60 and 120 min.

Combinations of different proteases have also been explored in relation to bitterness. Schlegel et al. [99] hydrolysed lupin protein isolate with various combinations of proteases, using either one or two step hydrolysis. Significantly higher bitterness compared with the protein isolate was not found for any of the combinations, with the exception of Alcalase and papain. Meinlschmidt et al. [100] hydrolysed soy protein isolate with various enzyme combinations, in one or two steps. Two combinations resulted in significantly higher bitterness compared with soy protein isolate—Alcalase, Neutrase, and Flavourzyme, and also for the same combination but with the addition of Corolase 7089. Interestingly, two of the combinations provided hydrolysates with a significantly lower bitterness than the protein isolate—Neutrase, Corolase 7089, and Flavourzyme, as well as the combination of papain and Flavourzyme.

The tendency towards bitterness of hydrolysates produced with Alcalase, or other subtilisins, is thought to be related to their broad specificity and preference for cleaving next to hydrophobic amino acid residues, which are then positioned as terminal residues in the peptides. Rezvankhah et al. [101] produced hydrolysates from lentil protein concentrate using either Alcalase, Flavourzyme, or a sequential hydrolysis with Alcalase followed by Flavourzyme. Bitterness, umami, and sweetness of the protein isolate and hydrolysates were assessed in an umami soup consisting of water, salt, and monosodium glutamate. Increased bitterness was only perceived for the Alcalase hydrolysate. Increased umami was apparent for all the hydrolysates, but particularly for the Alcalase and Alcalase/Flavourzyme hydrolysates. Increased sweetness was found for the Flavourzyme and Alcalase/Flavourzyme hydrolysates. It seemed that Flavourzyme was effective in eliminating the bitterness related to the Alcalase hydrolysis, as well as increasing sweetness perception. Although increased bitterness is by far the most prominent sensory impact of hydrolysis, other sensory attributes may also be affected to some extent. For example, Schlegel et al. [40] found that hydrolysis with pepsin significantly reduced the oatmeal-like retro-nasal attribute of lupin protein isolate.

Various approaches have been applied to reduce bitterness, which could be used in the preparation of pulse protein hydrolysates to improve sensory properties. These include complexation of bitter peptides with activated carbon, removal using hydrophobic column absorption, and exopeptidase treatment [96]. Application of exopeptidases is the most widely used approach for the debittering of hydrolysates. The terminal amino acids of peptides have a significant impact on bitterness; therefore removal of these residues with aminopeptidases or carboxypeptidases may allow reduced bitterness, while also retaining functionality [96,102]. At the same time, it is possible for a reduction in peptide length to contribute to reduced bitterness [96]. Ewert et al. [102] investigated the use of four different aminopeptidases for debittering a caseinate hydrolysate. These included three aminopeptidases, which they produced using *Lactobacillus* species, as well as Flavourzyme with its endoprotease activity reduced using ultrafiltration. Depending on the aminopeptidase used and its specificity, the treatment either reduced bitterness without impacting the functional properties, or improved functionality of the hydrolysate without affecting bitterness. In addition, release of free amino acids can increase the umami taste in hydrolysates. Großmann et al. [103] hydrolysed pea, soy, or canola protein using different commercial proteases which contain exoprotease activity. The specific activity of the proteases could be correlated to the free amino acid profile. Some significant differences were found between the hydrolysates for umami and bitter taste, depending on the protease. These properties also varied to some extent depending on the substrate.

In addition, the presence of other taste components may help to mask or reduce the perception of bitterness, such as umami or acids [33]; therefore, the acceptability of bitterness in hydrolysates may depend on the particular application. Bhaskar et al. [104] incorporated horse gram flour hydrolysate in the instant soup, and found that although panellists were able to identify the soup with hydrolysate from the control soup, there was no significant difference in preference between the two. Overall, various approaches can be taken to mitigate bitterness and improve sensory properties, especially careful selection of protease and control of hydrolysis, as well as application of exoproteases. It may be challenging to optimise the hydrolysis for both functionality and sensory quality simultaneously.

## 9. Future Outlook

Many studies have investigated enzymatic hydrolysis as a means of improving the functionality of pulse proteins, with varying results depending on different factors as previously discussed; however, further studies relating structural and surface properties to hydrolysate functionality would be useful. In addition, further research on factors such as pre/post hydrolysis treatments, ingredient processing, and their relationship to hydrolysate functionality should be carried out. In particular, work on the optimisation of enzyme inactivation conditions is largely missing from the literature; this could prove very useful in future studies regarding pulse protein hydrolysates. As pulse protein ingredients are increasingly being investigated and developed (e.g., with processing improvements), it is likely that more functional ingredients will be available and suitable for a range of applications; however, poor functionality of commercial ingredients may still be an issue, which is less evident in lab-scale ingredients, largely due to harsh processing conditions, leading to denaturation and aggregation. Enzymatic hydrolysis could potentially provide major functional improvements for such ingredients. Furthermore, there are many commercial protease preparations available that have not yet been investigated for the production of pulse protein hydrolysates, which could prove valuable. Moreover, novel techniques such as the ‘activity fingerprint’ have been described, which can provide rapid and detailed information on protease specificity using synthetic substrates; this could allow the prediction of hydrolysate composition and characteristics [32]. Technology such as protease engineering could provide even more options [105].

## 10. Conclusions

Enzymatic hydrolysis shows excellent potential as a tool for improving the functional properties of pulse protein ingredients where they are found to be lacking; however, it is difficult to predict the outcome of hydrolysis on ingredient functionality. Differences in protease specificity allow for a wide variety of peptides with differing properties in the hydrolysates. In addition, various other factors need to be considered, including protein substrate composition/structure, hydrolysis conditions/degree of hydrolysis, pre- and post-hydrolysis treatments, and target pH environment. Further investigations into the relationship between structure and surface properties and corresponding functionality will be useful. Improvements in solubility are often most effective where the protein substrate demonstrates poor initial solubility. In addition, enzymatic hydrolysis usually reduces the pH sensitivity of solubility, and the most significant relative increases are observed near the isoelectric point, which greatly increases the potential for the use of pulse proteins in acidic foods and beverages. Overall, in many cases, different functionalities can be improved with careful selection of proteases and control of hydrolysis. In addition, more research should be done on the effects of enzymatic hydrolysis on gelling properties as this is an essential functionality for many applications, and currently lacking in the literature. Furthermore, the effects of heat treatment and other treatments on functionality should be given more attention, especially in relation to food product processing. Sensory issues, especially bitterness, can be a limiting factor for the use of pulse protein hydrolysates. More work should be carried out to explore the relationship between hydrolysis and bitterness, specifically for various pulse proteins, along with debittering techniques. Overall, pulse protein ingredients will likely have an important role in satisfying global protein demand, in a more sustainable and economical manner. Modifications such as enzymatic hydrolysis can potentially be very useful as a means of increasing their utility, especially for plant-based food and beverage products.

## Figures and Tables

**Table 1:** Some commonly used proteases for food protein hydrolysis.

Enzyme Preparation	Main Activity	Origin
Alcalase	Serine endoprotease; broad specificity, preferentially hydrolyses peptide bonds at the C-terminal side of hydrophobic residues	*Bacillus lichenformis*
Trypsin	Serine endoprotease; specific for peptide bonds at the C-terminal side of Lys and Arg residues	Bovine/porcine pancreas
Chymotrypsin	Serine endoprotease; preferentially hydrolyses peptide bonds at the C-terminal side of Tyr, Phe, Trp and Leu residues	Bovine/porcine pancreas
Savinase	Serine endoprotease, broad specificity	*Bacillus lentus*
Protamex	Broad specificity endoprotease	*Bacillus* sp.
Corolase 2TS	Metallo endoprotease	*Bacillus thermoproteolyticus*, *Bacillus stearothermophilus*
Neutrase	Metallo endoprotease	*Bacillus amyloliquefaciens*
Pepsin	Aspartic endoprotease, broad specificity	Bovine/porcine gastric mucosa
Papain	Cysteine endoprotease, broad specificity	Papaya latex
Bromelain	Cysteine endoprotease, broad specificity	Pineapple stem
Flavourzyme	Exo and endoprotease mixture. Includes aminopeptidases, carboxypeptidases, and endoproteases	*Aspergillus oryzae*

**Table 4:** Overview of the effects of enzymatic hydrolysis on foaming properties from various protein sources and proteases.

Reference	Protein Source	Protease	Effect on Foaming Properties
Ahmed et al. [52]	Lentil protein isolate	Alcalase	FC: increased; FS: decreased
Al-Ruwaih et al. [51]	Kidney bean protein isolate	Alcalase	FC: increased (but decreased for high pressure treated sample) FS: decreased
Barać et al. [66]	Pea protein isolate	Chymosin	FC: increased at pH 3, 5, and 7; increased/decreased at pH 8 depending on HT FS: increased/decreased at pH 3 and 5 depending on HT; increased/no difference at pH 7 depending on HT; decreased at pH 8
Barac et al. [50]	Pea protein isolate (L1)	Papain	FC: increased at pH 3, 5, 7, and 8 FS: increased/decreased at pH 3 and 5 depending on HT; increased at pH 7; increased/decreased at pH 8 depending on HT
		*S. griseus* protease	FC: increased at pH 3 and 5; increased/decreased at pH 7 depending on HT; decreased at pH 8 FS: decreased at pH 3 and 5; no difference at pH 7; decreased at pH 8
	Pea protein isolate (Maja)	Papain	FC: increased at pH 3, 5, and 7; decreased/no difference at pH 8 depending on HT FS: increased at pH 3, 5, 7 and 8
		*S. griseus* protease	FC: increased/decreased at pH 3, 5, and 7 depending on HT; decreased at pH 8 FS: increased/no difference at pH 3 depending on HT; increased/decreased at pH 5 and 7 depending on HT; increased at pH 8
Betancur-Ancona et al. [63]	*P. lunatus* protein isolate	Alcalase	FC: decreased at pH 2, 4, 6, 8, and 10 FS: increased/decreased at pH 2 and 4 depending on HT; increased at pH 6 and 8; increased at pH 10
		Flavourzyme	FC: increased at pH 2, 4, 6, 8, and 10 FS: increased at pH 2, 4, 6, 8, and 10
Eckert et al. [39]	Faba bean protein isolate	Pepsin	FC: increased at pH 5 and 7 FS: no difference at pH 5; increased/no difference at pH 7 depending on HT
		Trypsin	FC: increased at pH 5; increased/no difference at pH 7 depending on HT FS: no difference at pH 5; increased at pH 7
		Flavourzyme	FC: increased at pH 5 and 7 FS: no difference at pH 5; increased at pH 7
		Neutrase	FC: increased at pH 5; increased/no difference at pH 7 depending on HT FS: increased/decreased at pH 5 and 7 depending on HT
Konieczny et al. [64]	Pea protein-enriched flour	Trypsin	FC: decreased at pH 4, 7, and 10 FS: decreased/no difference at pH 4 depending on DH; increased/no difference at pH 7 depending on DH; increased at pH 10
		Savinase	FC: decreased at pH 4, 7, and 10 FS: decreased/no difference at pH 4 depending on DH; increased at pH 7; increased/no difference at pH 10 depending on DH
		Papain	FC: increased/decreased at pH 4 depending on DH; decreased at pH 7 and 10 FS: no difference at pH 4; increased/no difference at pH 7 depending on DH; increased at pH 10
		Pepsin	FC: increased no/difference at pH 4 depending on DH; decreased at pH 7; decreased/no difference at pH 10 FS: increased at pH 4, 7, and 10
Schlegel et al. [40]	Lupin protein isolate	Alcalase	FC: increased; FS: no difference
		Papain	FC: increased; FS: no difference
		Neutrase	FC: increased; FS: no difference
		Protease N-01	FC: increased; FS: no difference
		Flavourzyme	FC: no difference; FS: no difference
		Protamex	FC: increased; FS: no difference
		Corolase 7089	FC: increased; FS: no difference
		Pepsin	FC: increased; FS: no difference
		Corolase N	FC: increased; FS: no difference
Yust et al. [42]	Chickpea protein isolate	Alcalase	FC: increased; FS: increased
Yust et al. [41]	Chickpea protein isolate	Flavourzyme	FC: increased; FS: increased/no difference depending on DH

This table is intended as an overview only—methodology, data representation, and statistics can vary between studies, making direct comparisons difficult. HT: hydrolysis time; DH: degree of hydrolysis; FC: foaming capacity; FS: foam stability.

## Data Availability

No new data were created or analyzed in this study. Data sharing is not applicable to this article.
